# Incorporating Monoclonal Antibodies into the First-Line Treatment of Classical Hodgkin Lymphoma

**DOI:** 10.3390/ijms241713187

**Published:** 2023-08-24

**Authors:** Theodoros P. Vassilakopoulos, Athanasios Liaskas, Patricio Pereyra, Panayiotis Panayiotidis, Maria K. Angelopoulou, Andrea Gallamini

**Affiliations:** 1 Department of Hematology and Bone Marrow Transplantation, National and Kapodistrian University of Athens, Laikon General Hospital, 11527 Athens, Greece; ath.liaskas@gmail.com (A.L.); ppanayi@med.uoa.gr (P.P.); mangelop@gmail.com (M.K.A.); 2 Department of Hematology, National Hospital Alejandro Posadas, Buenos Aires 1684, Argentina; patriciohernanpereyra@gmail.com; 3 Research and Clinical Innovation Department, Antoine Lacassagne Cancer Center, 06100 Nice, France; andreagallamini@gmail.com

**Keywords:** Hodgkin lymphoma, novel agents, first-line treatment, brentuximab vedotin, nivolumab, pembrolizumab

## Abstract

The long-term survival of Hodgkin lymphoma (HL) patients treated according to the current standard of care is excellent. Combined-modality schedules (ABVD plus radiotherapy) in early-stage disease, along with treatment intensity adaptation to early metabolic response assessed by PET/CT in advanced stage HL, have been the cornerstones of risk stratification and treatment decision-making, minimizing treatment-related complications while keeping efficacy. Nevertheless, a non-negligible number of patients are primary refractory or relapse after front-line treatment. Novel immunotherapeutic agents, namely Brentuximab Vedotin (BV) and immune checkpoint inhibitors (CPI), have already shown outstanding efficacy in a relapsed/refractory setting in recent landmark studies. Several phase 2 single-arm studies suggest that the addition of these agents in the frontline setting could further improve long-term disease control permitting one to reduce the exposure to cytotoxic drugs. However, a longer follow-up is needed. At the time of this writing, the only randomized phase 3 trial so far published is the ECHELON-1, which compares 1 to 1 BV-AVD (Bleomycin is replaced by BV) with standard ABVD in untreated advanced-stage III and IV HL. The ECHELON-1 trial has proven that BV-AVD is safe and more effective both in terms of long-term disease control and overall survival. Just recently, the results of the S1826 SWOG trial demonstrated that the combination nivolumab-AVD (N-AVD) is better than BV-AVD, while preliminary results of other randomized ongoing phase 3 trials incorporating anti-PD-1 in this setting will be soon available. The aim of this review is to present the recent data regarding these novel agents in first-line treatment of HL and to highlight current and future trends which will hopefully reshape the overall management of this disease.

## 1. Introduction

### 1.1. The Journey from Pathobiology to Novel Therapeutic Approaches

Hodgkin lymphoma (HL) is a lymphoid neoplasm defined by the presence of distinctive mononuclear and multinucleated Hodgkin/Reed–Stemberg (HRS) cells within the appropriate non-neoplastic tumor microenvironment (TME). The cellular origin of HRS cells remained largely unknown in the past due to their peculiar immunophenotype, namely the characteristic expression of CD15 and CD30 and the lack of expression of B-cell-specific surface antigens and CD45. However, microdissection studies performed directly on HRS cells and molecular studies including clonal rearrangement of the variable region of immunoglobulin heavy chain gene (IgVH) and single-cell RNA sequencing revealed that HRS cells originate from pre-apoptotic germinal center B-cells but with complete loss of their B-cell differentiation program [1]. This progress in single-cell isolation technology has allowed enormous progress in the understanding of lymphomagenesis and the pathophysiology of classical H (cHL). In 1994, Kuppers et al. from the Cologne group first showed the B-cell origin of HRS cells with a micromanipulation technique from lymph nodes of three patients [2]. Later on, two other important achievements came from the same Cologne group through HRS cell microdissection: Kanzler et al. were able to show somatic mutations in the VH gene amplified from HRS cells together with nonfunctional immunoglobulin (Ig) genes [3], and Schmitz et al. identified frequent somatic mutations in the TNFAIP3 (A20) gene in HRS cells, thus establishing these genes as tumor suppressors in the pathogenesis of HL [4].

CD30, first described in anaplastic T-cell lymphomas, is universally expressed by HRS cells [5] and soon became targetable [6]. Brentuximab Vedotin (BV) is an antibody-drug conjugate (ADC) consisting of an anti-CD30 IgG1 antibody and monomethyl auristatin E (MMAE), a microtubule-disrupting antimitotic agent. The conjugate targets CD30-positive HRS cells and induces microtubule inhibition, cell cycle arrest at G2/M phase and eventually apoptosis. BV was the first targeted agent approved for HL completely altering patients’ outcomes [7].

Another unique feature in terms of HL pathobiology is the paucity of HRS cells within the affected lymphoid tissue. In fact, HRS cells comprise only a small minority within a non-malignant inflammatory background of macrophages, T-cells, eosinophils, plasma cells and other immune cells forming a unique TME. Despite the immune milieu, HRS cells achieve immune evasion through various mechanisms and predominantly through the overexpression of programmed cell death-1 ligands (PD-L1 and PD-L2) caused by genetic alterations in the 9p24.1 locus. HRS cells also induce PD-L1 expression on macrophages. Interaction between PD-L1 and PD-1+ normal T cells in this immunoprotected “niche” leads to T-cell inhibition and promotes HRS cell survival. The interactions of PD-L1-positive tumor-associated macrophages (TAMs) with HRS cells and CD4+ lymphocytes have been pioneered by Carey et al. [8]. Thanks to multiplex immunofluorescence and digital image analysis, he described the spatial relationship of TAM with PD-1+ lymphocytes and HRS cells. Another cell interaction between CD4+/PD-1−/CTLA4+ cells on one side and HRS cells and TAMs on the other side, distinct from that engaging the PD-1/PD1-L1 blockade, seems to play an immunosuppressive role on CD4+ cells via interaction of CTLA4 with CD86 on HRS cells and TAMs, but the meaning of this needs further clarification [9]. The recognition of this crucial PD-L1 and PD-1 axis in the pathogenesis of HL paved the way for the development of two check-point inhibitors (CPIs), namely nivolumab and pembrolizumab. Their incorporation in the treatment of HL revolutionized patients’ survival in the relapsed/refractory setting and lately in the first line, as described in this review.

### 1.2. Treatment of Hodgkin Lymphoma in the Past Millennium: Lights and Shades

Thanks to the great progress in overall disease management during the last 50–60 years, not only early-stage but even advanced cHL has become a highly curable disease [10,11]. In early-stage disease, radiotherapy (RT) as monotherapy was gradually replaced by a combined-modality treatment (CMT) consisting of a brief or standard course of chemotherapy, ABVD in most cases, for 2, 3, 4 or 6 cycles, followed by 20–30 Gy limited field RT [12,13,14,15], in order to cope a low relapse rate with a reduced rate of long-term RT effects. However, increased awareness of the deleterious long-term RT sequelae paved the way to propose new trials aimed at investigating the effectiveness of chemotherapy (CT) alone vs. CMT [16,17]. During the last decade, positron emission tomography/computed tomography (PET/CT) performed early during (iPET) and after (EoT-PET) chemotherapy proved very useful to plan new PET-adapted strategies, with the aim of intensifying treatment only for the subset of patients with a poor prognosis, i.e., those with a positive iPET or EoT-PET. Four important, large, randomized PET-adapted clinical trials allowed researchers to reach the following conclusions [18,19,20,21]. Both in favorable and unfavorable early-stage HL with a negative iPET, CMT was statistically superior to CT alone, with a much more marked difference in favorable disease. However, due to the high effectiveness of rescue therapy, no difference in overall survival (OS) was recorded between CMT- and CT-alone arms. On the contrary, RT could be omitted without significant decremental disease control after 2 BEACOPP escalated plus 2 ABVD cycles, according to the German Hodgkin Study Group (GHSG) paradigm in early unfavorable disease [21].

In advanced-stage disease, following the initial success of MOPP achieved in the 1960s [22], the ABVD regimen, developed by Gianni Bonadonna in the 1970s [23], proved more effective and less toxic than MOPP and became, for a long time, the standard-of-care in advanced disease. While alternating and hybrid MOPP-ABV(D) regimens as well as Stanford V failed to overcome ABVD [24,25,26,27], the German strategy of treatment intensification in the form of BEACOPP-escalated chemotherapy finally provided a superior early- and long-term disease control at the expense of a considerably higher toxicity and a questionable survival benefit [28,29,30].

On the eve of the third millennium, the conundrum “ABVD or BEACOPP-escalated?” became less relevant with the introduction of early, interim PET/CT response assessment after two cycles of chemotherapy. After two ABVD cycles, nearly one-fifth of advanced-stage cHL patients have a positive iPET and derive a clear benefit from treatment intensification with BEACOPP escalated [31,32,33,34,35,36]. However, a non-negligible fraction of patients with a negative iPET (15–20%), who keep straight on with ABVD or AVD ultimately fail chemotherapy due to disease relapse or progression. On the other hand, after two cycles of BEACOPP escalated administered upfront, de-escalation to ABVD × 4 or only two further cycles of BEACOPP escalated in the case of a negative iPET, results in similar outcomes to those achieved with six fixed cycles of BEACOPP escalated [37,38,39], with 5-year progression free survival (PFS) rates as high as 86–90%. However, all patients are exposed to at least 2–4 cycles of BEACOPP escalated and a proportion of them to 6 cycles, with an overall expected toxicity higher than that recorded in patients undergoing BEACOPP intensification after a positive iPET on ABVD. At the time of this writing, it is still unclear which of the above strategies is preferable and whether a more aggressive treatment improves the long-term (OS), especially in the era of novel non-cytotoxic agents.

Another very important contribution to reduce the overall treatment toxicity in advanced-stage disease has come from the systematic use of EoT-PET/CT to assess the outcome of frontline therapy. Patients showing a non-fluorodeoxyglucose (FDG) avid (PET-negative) residual mass detected on CT after ABVD, randomized to receive consolidation RT on the residual mass or no further therapy, had exactly the same treatment outcome without a significant difference in terms of long-term disease control [36]. Similarly, the result of BEACOPP escalated without consolidation RT was excellent in patients without a residual mass or with a non-FDG-avid (PET-negative) residual mass, with 5-year PFS > 90%, and Kaplan–Meier PFS curves actually superimposable. Thanks to EoT-PET, RT was limited only to a small portion of patients (11%) showing an FDG-avid residuals mass with a diameter ≥ 2.5 cm in EoT-PET [40].

As cHL became more and more curable in the last three decades, the balance between treatment efficacy and toxicity turned out to be a crucial issue. In early-stage HL with a negative iPET, for example, the cure rate is very high, with the dilemma of omitting RT at the expense of a slightly inferior long-term disease control for patients treated with ABVD chemotherapy alone. More generally, in early-stage disease, the major issue is maintaining high cure rates, while minimizing the use of toxic regimens such as BEACOPP escalated when this regimen is administered either as treatment intensification after a positive iPET or as previously mentioned, in early-unfavorable patients treated with a radiotherapy-free regimen with two BEACOPP escalated cycles plus two ABVD cycles [21]. A similar situation is applicable in advanced disease. 

Starting from 2012, novel agents such as BV proved highly active in relapsed/refractory (r/r) cHL. Later, other monoclonal antibodies, the so-called CPIs, showed an even higher effectiveness in r/r cHL with long-term sustained responses. These agents have been gradually incorporated in first-line treatment, either as investigational or even as approved regimens, as discussed in detail in this review.

## 2. Randomized Trials Incorporating Novel Agents in the First-Line Therapy of Advanced-Stage Classical Hodgkin Lymphoma

### 2.1. The BV-AVD Combination: Incorporating Brentuximab Vedotin to ABVD

BV was evaluated in combination with standard ABVD in a phase 2 trial [41]. While the standard dose of BV monotherapy is 1.8 mg/kg every 3 weeks (not exceeding 180 mg, the dose corresponding to 100 kg), the administration of BV every 2 weeks in the backbone of ABVD was limited to 1.2 mg/kg. Additive pulmonary toxicity resulted in early toxic deaths, so that the omission of bleomycin became inevitable. Thus, BV-AVD was selected as the investigational immunochemotherapy combination to be compared with standard ABVD in the ECHELON-1 randomized trial. Patients with advanced disease, defined as Ann Arbor stage III/IV, with an Eastern Cooperative Oncology Group (ECOG) performance status 0–2 were enrolled in the phase III multicenter international clinical trial ECHELON-1, including 1334 patients, 64% of them being in stage IV. Patients were randomly allocated to receive a standard treatment with six ABVD courses, or the same regimen in which bleomycin was replaced by BV at the dose of 1.2 mg/Kg on day 1 and 15 of every cycle. The primary endpoint was the modified PFS (mPFS), in which the standard PFS events of disease progression/relapse or death of any cause were counted along with an additional PFS event defined as “a non-complete response with a Deauville 5-point scale score (D5PSS) of 3–5 according to Independent Review Committee (IRC) assessment, but only if followed by any antineoplastic therapy”. As ECHELON-1 met its primary endpoint of a superiority of the BV-AVD regimen with a 2-year mPFS rate per IRC of 82.1% vs. 77.2% in the standard (ABVD) arm, corresponding to a hazard ratio (HR) of 0.77 (*p* = 0.03), BV-AVD was approved by the U.S. FDA for first-line treatment of stage III/IV cHL [42]. A subgroup analysis according to a number of pre-specified risk factors revealed that the mPFS benefit was restricted to patients with stage IV, ≥1 extranodal site, high International Prognostic Score (IPS), etc. Consequently, the European Medicines Agency (EMA) and other national drug agencies released an approval for BV in first-line treatment of advanced cHL only to stage IV patients [42]. Quite recently, in the latest literature updates of the ECHELON-1 trial after a follow-up spanning beyond 6 years, the analysis was based on the standard PFS definition, and the PFS events recorded by the local investigators as Independent Review Facility (IRF) for central PET image review were no longer available beyond two years. The 6-year PFS rate was 82.3% vs. 74.5% for BV-AVD and ABVD, corresponding to a HR of 0.68 (95% CI 0.53–0.86) [43]. Interestingly, in a prognostic subgroup analysis, the superiority of the experimental arm, in terms of 6-year PFS, was demonstrated both in stage III and stage IV [43] (Table 1). More importantly, the 6-year OS rate was 93.9% vs. 89.4% corresponding to a statistically significant 41% reduction in the risk of death (HR 0.59; *p* = 0.009) [43]. Thus, ECHELON-1 was the first and unprecedented phase 3 randomized trial to show a statistically significant superior OS for a new frontline therapy regimen over ABVD, which had not been achieved even by BEACOPP escalated [30]. However, unlike PFS, the OS benefit was again restricted to patients with stage IV disease only. The results of efficacy and toxicity and the subgroup analysis of ECHELON-1 are summarized in Table 1. 

At the last analysis of the ECHELON-1 trial, in which the OS benefit was shown [43] the number of deaths was 39 and 64 with BV-AVD and ABVD, respectively. Toxic deaths during treatment were 9 with BV-AVD [7/9 due to febrile neutropenia in patients not receiving primary Granulocyte Colony Stimulating Factor (G-CSF) prophylaxis] vs. 13 during ABVD (11/13 due to pulmonary toxicity, probably bleomycin-related) for an overall toxic death rate of 1.4% vs. 1.9%. Deaths due to HL or complications were 32 vs. 45, while only 1 patient in the BV-AVD arm died of a second malignancy vs. 11 in the ABVD arm [43]. The routine use of primary G-CSF prophylaxis might have reduced the toxic deaths in the BV-AVD arm, while pulmonary deaths probably would have been reduced in the ABVD arm if the paradigm adopted in the RATHL trial had been applied and bleomycin had been omitted following a negative iPET [33]. Finally, as patients were enrolled in ECHELON-1 between November 2012 and January 2016, it is not clear if all patients with r/r HL after treatment in ECHELON-1 had the opportunity to receive PD-1 inhibitors and thus enjoy a prolongation of OS.

If BV-AVD is chosen as frontline treatment of advanced-stage cHL, a crucial point is to administer primary G-CSF prophylaxis, as recommended by the Independent Data and Safety Monitoring Committee due to the higher incidence of febrile neutropenia in the BV-AVD arm (19% vs. 11%). This measure reduced the incidence of febrile neutropenia and was established as a recommendation for clinical practice. Hospitalizations were more frequent with BV-AVD. Toxicity profiles were different for BV-AVD and ABVD; neutropenia (including febrile neutropenia), peripheral neuropathy (PN) and diarrhea were more common with BV-AVD, while pulmonary toxicity was more common and potentially lethal with ABVD [42]. Very importantly, up to 40% of the patients in the experimental arm suffered from sensory or motor PN; among them, 5% had a grade 3 sensory neuropathy, vs. 21% and <1%, respectively, in the standard arm. Finally, 125 of 662 patients (18.9%) in the BV-AVD group and 59 of 659 (9.0%) in the ABVD group had ongoing PN at the last follow-up.

A special mention is deserved for the prognostic/predictive value of iPET on treatment outcome, which was not included in the prespecified prognostic group analysis of the ECHELON-1 trial but was evaluated in the 5-year follow-up analysis [44]. The positive iPET (D5PSS 4–5) rate was 7.4% and 9.1% in the BV-AVD and ABVD arm, respectively, which was surprisingly low compared with the 16–24% rate expected with ABVD for advanced cHL [30]. Despite the observed superiority of the experimental vs. standard arm, both in iPET-negative and positive cohorts, in terms of statistical significance, it should be noted that this superiority was demonstrated in iPET-negative patients thanks a to an eleven-times larger cohort of patients compared to that of iPET-positive patients (1166 vs. 105). The ECHELON-1 trial underlines once again the adverse prognostic meaning of a positive iPET whatever the treatment chosen. As a matter of fact, in the standard and experimental arms, the 5-year PFS was 78.9% and 84.9% for iPET-negative (+6.0%) vs. 45.9% and 60.6% (+14.7%) for iPET-positive patients, respectively (Table 1) [44]. Overall, continuing BV-AVD after a positive iPET is an acceptable strategy based on the 60.6% PFS rate at 5 years. However, this result could not be compared to that obtained upon switching to BEACOPP escalated as in the RATHL [33] or HD0607 trial [34], as patients with score 5 enrolled in the ECHELON-1 trial, both in the experimental (*n* = 21: 4%) and the standard arm (*n* = 30: 5%), were allowed to receive a salvage regimen, and this was not counted as an event. Interestingly, the ECHELON-1 results further confirm the suboptimal negative predictive value (NPV) of iPET after ABVD, which fell into the expected range for stage III/IV patients [30,33,35,46,47] and was only marginally better with BV-AVD, as 15% of iPET-negative patients still progressed or relapsed even with the incorporation of BV [44].

The question of whether treatment escalation would be effective in patients remaining iPET-positive after BV-AVD is being preliminarily addressed by the COBRA (EORTC-1537), a single-arm phase 2 study exploring the significance of early metabolic response after as few as one cycle of BV-AVD. All the patients received one cycle of BV-AVD followed by iPET. Patients with a negative iPET continued with five additional BV-AVD cycles, while iPET-positive patients were escalated to BreCADD—a BV-containing but less toxic variant of BEACOPP escalated (see below)—for six cycles. Researchers also measured thymus and activation-regulated chemokine (TARC) levels both at baseline (bTARC) and after one cycle of BV-AVD (iTARC). A serum iTARC level > 1000 pg/mL was considered positive for detecting active disease after one cycle of BV-AVD. With 150 patients enrolled in the study, iPET at one cycle was positive in 40% of them. Within the group of patients with available iTARC and positive bTARC levels (*n* = 84), iPET was positive in 33 cases (39%) while iTARC was positive in 12 cases (14%) only, among whom 8 were also iPET-positive. These figures suggest a high rate of false positive results for iPET after a single course of BV-AVD. Researchers concluded that the majority of advanced-stage HL patients showed a treatment response already after one cycle of BV-AVD, as measured by FDG-PET and serum TARC [48].

### 2.2. Novel Combinations with Brentuximab Vedotin and Checkpoint Inhibitors: Moving beyond BV-AVD

The 6-year PFS assessed by investigators in ECHELON-1 was just above 82% with BV-AVD, being 84.4% for patients < 60 years old, who would be eligible for BEACOPP escalated (vs. 77.1% with ABVD) [43]. Although this figure approaches the 86–90% obtained with the PET-adapted BEACOPP-escalated approaches [37,38,49], it still appears somewhat lower. By replacing vincristine and bleomycin with BV in the more aggressive BEACOPP-escalated polychemotherapy, the GHSG proposed the new BreCAPP regimen to avoid synergistic neurotoxicity and pulmonary toxicity. In the BreCADD regimen, further modifications were adopted to reduce toxicity: Dacarbazine replaced procarbazine in order to reduce genotoxicity and leukemogenicity, and the 14-day prednisone course was replaced by a 4-day dexamethasone course in order to avoid prolonged steroid administration, especially during severe neutropenia. BreCAPP and BreCADD were compared in a randomized phase 2 trial [50,51]. The efficacy was similar with both regimens, but BreCADD was selected for further development due to its more favorable toxicity profile.

The HD21 randomized trial of the GHSG attempts to compare the original BEACOPP-escalated regimen with BreCADD, given for four or six cycles according to the results of iPET, upon enrollment of 732 and 738 patients in each group, respectively. Primary endpoints were non-inferiority of PFS and the reduction of treatment-related morbidity (TRMB); namely any grade 3–4 organ toxicity or grade 4 hematologic toxicity (anemia, thrombocytopenia, infections). TRMB was documented in 59% vs. 42% of patients in the BEACOPP-escalated and BreCADD arm, and hematological TRMB events were documented in 52% vs. 31%, respectively (*p* < 0.001). Of note, only 8% and 6% of patients needed at least one red cell and platelet transfusion in BreCADD group, compared to 22% and 13% in patients who received BEACOPP-escalated. TRMB organ toxicity did not differ significantly between the two arms [52]. The results from the interim efficacy analysis at 40 months were rather impressive. Early PFS events at 1-year were notably reduced in BreCADD arm (2.2% vs. 5.0%), and the 3-year PFS was 94.9% vs. 92.3% for the BreCADD and BEACOPP groups, respectively. OS at 3 years was 98.5% for both arms [53]. Overall, the BreCADD regimen offered a significant and clinically relevant reduction of TRMB and outstanding efficacy in terms of PFS compared to BEACOPP escalated in patients with newly diagnosed advanced cHL. However, these results may not reflect the efficacy of BreCADD in the whole spectrum of patients with advanced-stage disease, as the study population had an upper age limit of 60 years and also included patients with stage IIB with bulky or extranodal disease, falling within the definition of advanced-stage disease according to GHSG. Furthermore, BreCADD is associated with less toxicity than BEACOPP but may still be more toxic than ABVD and equivalent regiments. 

The results of the S1826 SWOG trial appeared even more provocative and revolutionary. SWOG S1826 included 994 patients with advanced-stage cHL, defined as stage III/IV, who were randomized to receive BV-AVD or the combination N-AVD (nivolumab-AVD). Of note, the study had no upper age limit and included children and adolescents ≥ 12 years old and 1.5% well-controlled HIV-positive cases but also excluded patients with interstitial lung disease and/or pneumonitis and—more importantly—those with active autoimmune disease. Thus, ~25% of the patients were younger than 18 and ~10% older than 60 years old, patients who would not have been included in any BEACOPP-based trial. In terms of safety, neutropenia was more common in the N-AVD arm. This is rather unexpected, but G-CSF was more frequently used in the BV-AVD arm (54% vs. 95%), probably because of the already established clinical experience, and also resulted in more frequent bone pain. Interestingly, this discrepancy probably explains the slightly higher frequency of febrile neutropenia, infection/sepsis, anemia and thrombocytopenia in the BV-AVD arm. PN, predominantly sensory, was clearly more common in patients who received BV-AVD, and the incidence of immune-related events was overall low and mostly of grade ≤ 2 in the N-AVD group. Notably, liver and pulmonary toxicity as well as colitis were similar between the two arms. Discontinuation of BV was twice as frequent compared to discontinuation of nivolumab (22% vs. 11%). The difference between the two groups in 1-year PFS was remarkable: 94% for patients treated with N-AVD and 86% for those who received BV-AVD [45]. A summary of the results on safety and efficacy in comparison with the ECHELON-1 trial are presented in Table 1. Even at 3 years, OS is ~97% and ~99% in the BV and nivolumab arms with 11 vs. 4 deaths recorded and only 1 disease-related death in a patient who actually never received treatment. Toxic deaths were minimal and clearly <1% in the N-AVD arm. 

Based on these exciting data, the off-label use of nivolumab in stage III/IV patients or the off-label use of BV within BreCADD in stage III patients become very attractive. N-AVD appears better than BV-AVD but it is not clear if nivolumab synergizes with AVD to obtain a greater tumoricidal effect or mainly delays the development of relapse. N-AVD has not been compared with BreCADD. Its efficacy may have been augmented by the inclusion of 25% young patients <18 years old or reduced by the inclusion of 10% older patients who are not eligible for BreCADD. However, the very favorable toxicity profile of N-AVD probably renders this regimen more attractive for everyday use. Furthermore, adopting N-AVD may provide a less complicated way to treat advanced cHL by reducing or eliminating the need of an interim PET. Further follow-up is needed both for SWOG S1826 and HD21 to accurately estimate the efficacy and long-term toxicity of N-AVD and BreCADD. 

## 3. Brentuximab Vedotin in the First-Line Therapy of Early-Stage Classical Hodgkin Lymphoma

According to the RAPID, EORTC H10 and GHSG HD16 trials, RT cannot be omitted without avoiding a rather small percentage of progressions/relapses in patients with early favorable, non-bulky HL despite a negative iPET after two cycles of ABVD (5–10% additional cases) [18,19,20]. Thus, in the large subgroup of iPET2-negative patients, the omission of RT after risk-stratified administration of 2–6 cycles of ABVD results in a 5-year PFS is as high as 85–90%. The outcomes are similar in iPET-negative patients with early unfavorable disease after six cycles of ABVD without RT [18]. In addition, depending on D5PSS cutoff, a significant proportion of early-stage patients remain iPET-positive and experience inferior outcomes even after RT [18,19,20]. 

The rationale of incorporating BV in the treatment of localized-stage HL includes the elimination of the significant long-term toxicity of RT and the pulmonary toxicity of bleomycin, while maintaining and even improving survival. Several prospective small and medium-sized phase 2 trials which have enrolled patients with favorable and unfavorable (or early and intermediate) disease with variable inclusion of bulky disease have been published/reported. Most of them have adopted an iPET-driven strategy to determine treatment intensity and have incorporated BV into an AVD or AD backbone, omitting bleomycin or even vinblastine and eliminating RT or limiting RT to patients with residual disease at the EoT. A single study evaluated the consolidation with six BV cycles after ABVD. The major characteristics and outcomes of these studies are summarized in Table 2.

Abramson et al. evaluated BV-AVD in a small series of 36 patients with favorable and non-bulky unfavorable disease [54]. In a subsequent study of 34 patients with similar profile, vinblastine was further omitted resulting in the BV-AD combination [55]. BV-A(V)D was administered for four or six cycles according to iPET-2 results. As summarized in Table 2, the rate of iPET2 positivity was 0% and 6% respectively; thus, the majority of patients received four A(V)D cycles. PFS was 94% at 3 years and 91% at 5 years in the two trials, and OS was 96–97%. BV-AVD was associated with higher rates of grade 3 PN and neutropenic fever (24% and 29%, respectively) compared to clinical experience with conventional ABVD, but these side effects were no longer observed with BV-AD despite the lack of preemptive G-CSF use. Overall, PN was still noted in 59% of patients receiving BV-AD, but only three patients developed grade 2 and none grade 3 events [55]. Interestingly, although a previous attempt of omitting two cytotoxic drugs from the ABVD combination in the setting of two chemo cycles plus RT has failed [59], the substitution of both bleomycin and vinblastine with BV appears promising and could be further explored as a potential first-line combination in an RT-free approach.

Another very interesting, flexible approach was proposed by Park et al., who explored the role of a 6-cycle BV consolidation strategy as a substitute for RT in a phase 2 study enrolling 39 patients with localized favorable and non-bulky unfavorable disease. Prior to BV consolidation, they received 2–6 cycles of ABVD in a risk- and iPET2-based stratified approach [56]. Since D5PSS-3 was considered a positive iPET2 result, 28% of the patients had a positive iPET and received more cycles of ABVD; however, only 5% received RT based on a positive EoT-PET, as 37/39 had negative EoT-PET. This strategy followed by six cycles of BV consolidation and associated with a treatment adaptation both on iPET2 and EoT-PET, resulted in a 3-year PFS of 92%, which was 100% for the EoT-PET-negative patients with a minimal percentage of patients receiving RT, and only 2.5% for patients complaining of a grade 3 PN (Table 2). 

In a larger study, with the aim of assessing the ability of BV to replace consolidation RT in localized stages, including in patients presenting at baseline with a bulky disease, Kumar et al. enrolled 116 patients with intermediate (early unfavorable) stage HL according to the GHSG criteria, in a four-cohort, non-iPET-adapted study starting with four cycles of BV-AVD followed by RT in various dose and field schedules (cohorts 1–3; 88 patients) or no RT at all (cohort 4; 29 patients) [57]. Of note, 86% of patients had a large nodal mass (≥7 cm), 27% had classical bulky disease per traditional definition (≥10 cm) and 23% had stage IIB with bulky or extranodal disease and were thus considered as advanced stage according to GHSG. Notably, all patients in the no-RT cohort 4 had at least nodal masses ≥ 7 cm. As summarized in Table 2, the rate of iPET-2-negativity was 87%, while EoT-PET-4 was negative in 93–100% of the patients. Very interestingly, the 2-year PFS were similar in cohorts 1–3 treated with consolidation RT (93–100%) and in RT-free cohort 4 (96.6%) in evident contrast with the impaired outcome of favorable early-stage patients with nodal masses ≥ 5 cm and a negative iPET after ABVD × 3 enrolled in the RAPID trial [19,60]. The rates of serious PN and febrile neutropenia were low. Very importantly, a recent update after an extended 4-year follow-up period showed sustained long-term disease control even in the RT-free cohort, with eight progression events and two deaths and an overall 4-year PFS and OS of 93% and 98%, respectively for patients treated with BV-AVD alone. A combination of iPET-2 (D5PSS 1–3 vs. 4–5) and the baseline metabolic tumor volume (MTV) (< or >150 mL) group defined a small subgroup of patients with high baseline MTV and a positive iPET-2 with a 4-year PFS of 60% vs. 95–100% for all other combinations (*p* = 0.001) [61].

All these phase 2 studies provided very promising results compared to those expected with an iPET-guided approach to spare RT. However, none of them was designed as a classic randomized phase 3 trial. BREACH is the only randomized phase 2 trial reported so far comparing BV-AVD plus RT to classical combined-modality with ABVD plus RT in patients with early unfavorable HL, i.e., those with ≥1 EORTC risk-factors (Table 2) [58]. With the goal of verifying whether BV-AVD leads to a higher iPET-2 negativity rate compared to classical iPET-2 during ABVD (with an increment of at least 10% compared to that obtained in the H10 trial), 170 patients were enrolled and randomized in a ratio of 2:1 to receive BV-AVD or ABVD and 30 Gy consolidative RT. The rate of iPET-2 negativity (D5PSS < 4) was 82.3% in the BV-AVD vs. 75.4% in the ABVD arm. Overall, after combined modality therapy, the 2-year PFS was higher in the BV-AVD vs. standard ABVD arm (97.3% vs. 92.6%), and the results were similar among iPET-2-negative patients reaching a 2-year PFS of 97%. In contrast, the difference was more profound in iPET-2-positive patients, as the 2-year PFS was 93.8% for BV-AVD patients vs. 71.6% for ABVD. Thus, iPET was no longer predictive of treatment outcome during BV-AVD+RT, while it was after ABVD+RT. Despite the randomized approach, RT was kept in both arms, thus giving no answer on the question whether consolidation RT could be omitted in early unfavorable HL treated with BV-AVD. Notably, the baseline MTV was also assessed in the BREACH study and turned out to be the most important predictor of treatment outcome, even superseding BV-AVD and iPET-2: a high total metabolic tumor volume (TMTV) (>147 mL) was associated with a significantly shorter 2-year PFS (90.9% vs. 70.7%, HR: 17.9). PFS was similar in patients with low MTV with either regimen, but a more apparent difference was noted among patients with high TMTV (>147 mL) (90.9% vs. 70.7% for BV-AVD and ABVD arms, respectively). 

Overall, the pros and cons of the above studies support the idea to conduct a randomized trial comparing BV-AVD (or BV-AD in selected patients) vs. an ABVD-based iPET2-guided treatment in localized HL. 

## 4. Checkpoint Inhibitors in the First-Line Therapy of Classical Hodgkin Lymphoma

The CheckMate-205 and KEYNOTE-087 trials are widely known for the outstanding results of the CPIs nivolumab and pembrolizumab in the treatment of r/r cHL, which completely overturned the dismal outcome of these patients [62,63,64,65,66,67,68,69,70]. Several new studies are ongoing at the time of this writing and aim to explore the effectiveness of the incorporation of CPIs in the first-line treatment of cHL. Complementing the outstanding SWOG S1826 trial, which was previously analyzed in detail, some of them are addressed below and summarized in Table 3. Generally, these studies include a lead-in part with a CPI monotherapy followed by AVD or a CPI-AVD combination. Bleomycin was completely abandoned because of concern about synergistic pulmonary toxicity, which is also a common side-effect of CPIs. The lead-in part of these trials is very informative regarding the efficacy of CPI monotherapy in the setting of previously untreated cHL. Although advanced-stage disease was the main target of such studies, an admix with localized stages was permitted in some of them. However, the exact figures of ORR and CR rates as indicators of treatment outcome should be viewed with caution for two main reasons: (1) because the reference population (the denominator) may change due to some dropouts mainly for toxicity and (2) inconsistency of the standard criteria to assess treatment response after CPI administration.

**Figure 1:**
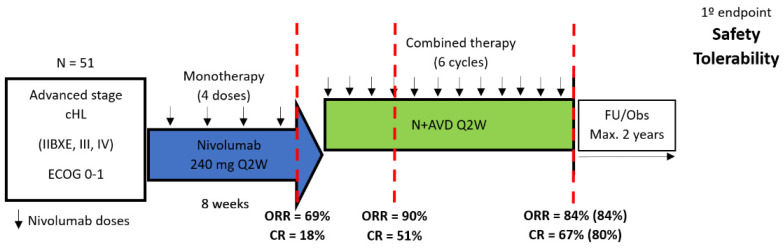
Study design and response rates of Phase II CheckMate 205 Study (cohort D). cHL = classical Hodgkin lymphoma; ECOG: Eastern Cooperative Oncology Group; FU/obs = follow-up/observation, Q2W = once every two weeks, N+AVD = nivolumab, doxorubicin, vinblastine, dacarbazine, CR = complete responses; ORR = overall response rate (numbers in parentheses refer to rates per investigator). It should be noted that response assessments were made by using 2007 International Working Group criteria [75] and not the revised criteria of Lugano [76].

The well-known CheckMate-205 study included a cohort D of patients with newly diagnosed, advanced-stage HL and aimed to evaluate the efficacy of nivolumab monotherapy followed by the combination of nivolumab plus AVD [71]. That arm included 51 patients with advanced-stage disease, the latter defined as stage III-IV or IIB with bulky or extranodal disease. In the lead-in part of the study, patients received nivolumab for four infusions and then six cycles of AVD. At the end of monotherapy, ORR was 69%, with 18% CRs, but this was evaluated based on the strict 2007 nternational Working Group (IWG) criteria, in which D5PSS-3 and some D5PSS-2 cases are classified as positive [75]. Per Independent Review Committee (IRC), the ORR and CR rates at EoT were 84% and 67%, but 5/51 patients were not evaluable for response and were classified as “non-responders” (Figure 1). Among the 46 evaluable patients, ORR and CR rates were 93% and 74%. Interestingly, as assessed by the investigators, the CR rate was higher at 80% but still low compared with the 9-month PFS and OS of 92% and 98%, respectively, with the apparent contradiction of a suboptimal percentage of interim and EoT responses (Figure 1) against a high long-term durable complete disease control without recurrence. This probably reflects the validity of the Lugano 2014 of EoT-PET/CT interpretation vs. the 2007 IWG criteria, “logistic issues” regarding the statistical handling of dropouts and probably a degree of uncertainty of EoT-PET/CT interpretation after CPI therapy. The study design and the response rates at various time-points are depicted in Figure 1. Overall, 59% of patients experienced grade 3–4 treatment-related adverse events with the most common being neutropenia (49%), including 10% febrile neutropenia. Immune-mediated adverse events were all grade 1–2 except for two cases (4%) of grade 3 hepatitis requiring high-dose steroids. A single treatment-related death was reported (2%) due to febrile neutropenia and complications in a 68-year-old patient. CheckMate-205 cohort D highlighted for the first time that the sequential combination of nivolumab plus AVD is feasible and effective. Moreover, it should be stressed that the mentioned inconsistency of the standard criteria with FDG-PET/CT in the evaluation of treatment response in lymphoma [76] to assess the response to CPIs has not been dissipated by the publication of specific metrics to measure CPI response entitled “refinement of Lugano Classification lymphoma response criteria” by adopting the oxymoron “criteria to define an indeterminate response”, the so-called LYRIC criteria [77].

**Figure 2:**
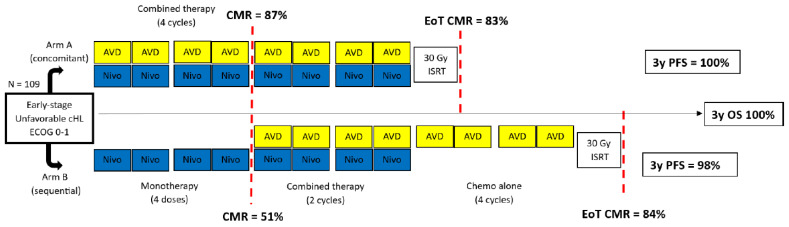
Study design and response rates of NIVAHL. cHL = classical Hodgkin lymphoma; ECOG: Eastern Cooperative Oncology Group; Nivo = nivolumab, AVD = doxorubicin, vinblastine, dacarbazine, EoT = end of treatment, CMR = complete metabolic response; ISRT = involved-site radiation therapy, 3y PFS = 3-year progression-free survival.

NIVAHL was a randomized phase 2 study that explored the efficacy and the safety of nivolumab plus AVD and consolidation RT in 109 patients with “intermediate” stage cHL (early unfavorable localized), as defined by GHSG [78]. Patients were randomized in two groups based on two different treatment schedules, both containing eight nivolumab doses in total: concomitant treatment with nivolumab plus AVD (N-AVD) for four cycles, or sequential treatment starting with four infusions of nivolumab at 14-day intervals, followed by two N-AVD and two AVD cycles (Figure 2). After chemo-immunotherapy, all patients received consolidative RT. As expected during standard ABVD treatment, a high early metabolic CR (CMR) rate of 87% was observed at iPET2 after two N-AVD cycles; interestingly, however, more than half (51%) of the patients who received four infusions of nivolumab alone also achieved an early CMR. The latter is in sharp contrast to the 18% CMR rate observed in the lead-in phase of the CheckMate-205 study cohort D [71] (Figure 1) and can be explained by the different set of criteria for response assessment used in the two studies [75,76]. At the end of systemic therapy, i.e., after the same total treatment burden, the CR rate was similar in the two cohorts (83% and 84%). Median follow-up was 41 months. Once more, despite the <85% CR rate at EoT, the 3-year PFS was estimated at 98% and 100% in the sequential and concomitant treatment group, respectively, and the 3-year OS was 100% [72]. However, it should be noted that the impressive results seen in the NIVAHL study were achieved with universally given RT after immunochemotherapy.

**Figure 3:**
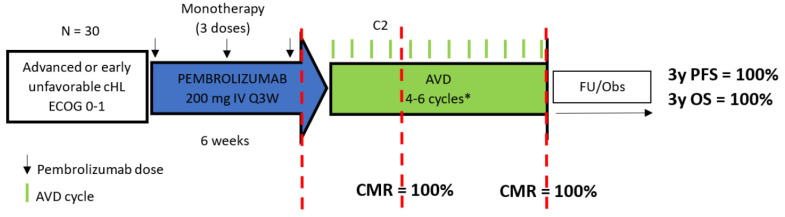
Treatment schedule and response rates of sequential administration of pembrolizumab–AVD. cHL = classical Hodgkin lymphoma; ECOG: Eastern Cooperative Oncology Group; AVD = doxorubicin, vinblastine, dacarbazine, CMR = complete metabolic response; FU/obs = follow-up/observation, 3y PFS = 3-year progression-free survival; 3y OS = 3-year overall survival, *, depending on patient stage and disease bulk.

**Figure 4:**
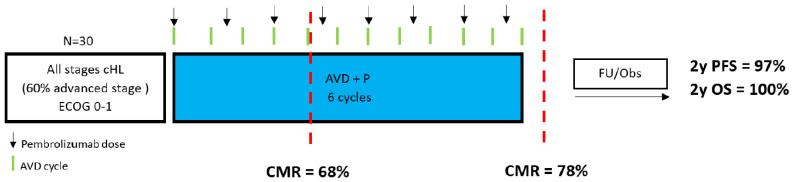
Treatment schedule and response rates of concomitant administration of pembrolizumab–AVD. cHL = classical Hodgkin lymphoma; ECOG: Eastern Cooperative Oncology Group; AVD + P = pembrolizumab, doxorubicin, vinblastine, dacarbazine, CMR = complete metabolic response; FU/obs = follow-up/observation, 2y PFS = 2-year progression-free survival; 2y OS = 2-year overall survival.

Similarly, Allen et al. and Lynch et al. evaluated in phase 2 trials the combination of pembrolizumab and chemotherapy in sequential and concurrent schedules, respectively (Figure 3 and Figure 4). Sequential pembrolizumab plus AVD was given to 30 patients with advanced or early unfavorable disease, as defined by the NCCN criteria. Patients received three infusions of a fixed dose of pembrolizumab every 3 weeks followed by 4–6 cycles of AVD, depending on patient stage and disease bulk. Metabolic response was assessed after pembrolizumab monotherapy, after two cycles of AVD and at EoT. At the end of the single agent pembrolizumab, a CMR was observed in 37% of patients and an additional 23% achieved near CMRs defined as ≥90% reduction from baseline MTV. The CMR rate after two AVD cycles was 100% and remained at this percentage at EoT [79]. After a median follow-up of approximately 3 years, none of the patients relapsed, with PFS and OS reaching 100% [73]. This study highlighted the potential of obtaining a CMR after a short course of pembrolizumab even in patients with bulky disease, which consisted of the 40% of study population. 

Lynch et al. assessed the effectiveness of pembrolizumab administered concurrently with AVD (AVD + P) in another single-arm study of 30 untreated, all-stage HL patients (60% with advanced disease and 20% early unfavorable per NCCN criteria) [74,80]. According to the Lugano criteria, after two cycles of AVD + P, 68% of the patients were iPET2-negative, while 78% had a negative EoT-PET after two to six AVD + P cycles (two, four and six cycles in 17%, 10% and 73% of the patients). Although these “metabolic” results in interim and EoT-PET appear inferior to those achieved with ABVD alone [30,33,36,81], no iPET-2-positive patient and only one out of five patients with a positive EoT-PET (out of 23 evaluable), experienced treatment failure. Three patients with early favorable disease received preplanned consolidation RT. There were no treatment-related deaths, and the rate of febrile neutropenia was 17%. Of note, at least one dose was missed or pembrolizumab treatment was interrupted in 20% of patients due to toxicity, while 10% of the patients developed temporary and well-manageable grade ≥ 3 transaminitis, leading to pembrolizumab discontinuation. None of the patients who stopped treatment with pembrolizumab due to toxicity has relapsed yet [80]. With a median follow-up time of 2.1 years, the 2-year PFS and OS were 97% and 100%, respectively [74]. 

Finally, Keynote-C11 is another phase 2 study aiming to assess the efficacy of pembrolizumab plus chemotherapy based on an iPET-based approach. Eligible participants are patients with early unfavorable and advanced stage disease. All patients will undergo PET/CT (PET3) after three infusions of pembrolizumab and two subsequent AVD cycles (phase 1). Patients with a negative PET3 will continue with 2–4 cycles of AVD (phase 2) depending on stage and disease bulk, whereas patients with a positive PET3 will receive 2–4 cycles of BEACOPP escalated if up to 60 years old or 4 AVD cycles for patients >60 years. All patients will receive four cycles of pembrolizumab at a dose of 400 mg every six weeks as consolidation therapy, which corresponds to eight standard pembrolizumab cycles. Until now, 146 patients have been included with a median age of 34.5 years; 20% of patients had bulky disease, while 55% and 42% had advanced and early unfavorable disease, respectively. At data cut-off, the median follow-up was only 3.2 months with 32% of the patients being on pembrolizumab monotherapy, 29% being on chemotherapy phase 1, 23% on chemotherapy phase 2 and 7% on pembrolizumab consolidation. Grade ≥ 3 drug-related adverse events were reported in 14% of patients who received pembrolizumab monotherapy or consolidation, 54% of patients who received AVD, and in one of two patients who received BEACOPP escalated. There were no deaths due to drug-related events. Immune-mediated events were reported in 19% of patients who received pembrolizumab monotherapy or consolidation. Obviously, no efficacy results are available yet [82].

In addition to the CPI nivolumab and pembrolizumab, avelumab is a PDL1 inhibitor approved for treatment of patients with urothelial and renal cell carcinoma, which was also active in a phase 1b trial of r/r cHL [83]. The AVENuE window study aimed to assess the efficacy of avelumab prior to a PET-adapted treatment strategy. Overall, 47 patients with stage III/IV or high-risk stage II disease (B, bulk or ≥3 nodal sites) were enrolled. All patients enrolled received four infusions (two cycles) of 10 mg/kg avelumab every 2 weeks followed by PET/CT (PET1) and two additional cycles of ABVD followed by a second PET/CT (PET2). PET2-negative patients (Deauville score 1–3) received four cycles of AVD afterwards, whereas PET2-positive patients were treated with four cycles of escalated BEACOPP. The RATHL trial was used as a comparative historical cohort [33]. The primary endpoint was ORR after two cycles of avelumab, with a >40% rate deemed worthy of further study. The ORR at PET1 was 45%, including 11% CMR. At PET2, the positivity rate was 10.6% compared to 16.3% in RATHL, despite the much higher percentage of stage IV in the AVENuE study. With a median follow-up of 14 months, the 1-year PFS was 100% but two patients progressed within 23 months. Grade 3/4 avelumab-related adverse events were reported in nine patients [84]. Avelumab could be a promising agent, but more follow-up is needed to define its efficacy. 

Summarizing these data, the SWOG S1826 trial provided a new treatment paradigm for advanced cHL, which is also applicable in elderly patients, and appears less toxic than BEACOPP-based approaches and more effective than BV-ABVD—and consequently ABVD—at least in the short term. Despite these considerations, the nivolumab-AVD combination has not been approved yet. Despite this great success, several questions still remain open. The various combinations of all CPIs with conventional chemotherapy have led to very promising results with impressive PFS and OS rates, which need to be validated in the long term. Immune-mediated toxicity remains an issue, although limited to a relatively small number of patients. However, toxicity and potential relative or absolute contraindications (cardiac and pulmonary reserves, active autoimmune background) are of special concern especially in this treatment-naïve population. More studies are needed in order to define the optimal combination and treatment schedule, the role of PET-adapted strategies and circulating tumor DNA, the potential application of CPI-chemo combinations in early-stage disease and the optimal amount of treatment required in each setting. With the increasing success rate of CPI–chemotherapy combinations, conventional and biologic prognostic factors should be redefined further [85,86,87]. Whether circulating tumor DNA and PDL-1 expression can determine response to such combinations also needs to be further assessed [71,74]. 

## 5. Brentuximab Vedotin and Checkpoint Inhibitor Combinations in the First-Line Treatment of Classical Hodgkin Lymphoma

Another approach with a slightly different rationale consists of the combination of two novel agents in a chemotherapy-inclusive treatment schedule. 

SGN35-027 is a phase 2 single-arm study in three parts: part A aims to explore the efficacy and safety of the combination BV-AVD when administered with growth factor prophylaxis; part B evaluates the efficacy and tolerability of the combination of BV, nivolumab, doxorubicin and dacarbazine (AN+AD) in patients with disease stage III/IV or stage I/II with bulky mediastinal mass; and part C focuses on the efficacy and tolerability of AN+AD in patients with stage I/II non-bulky cHL. The primary objective of parts B and C was to estimate the CMR rate at EoT in previously untreated patients with HL. The preliminary results of the SGN35-027 study have been presented. In part B, 57 patients were enrolled with a median age of 35 years; 51% had stage IV disease. The overall CMR rate was 88% at EoT. With a median follow-up of 15.1 months, 7% had progressive disease and 2% died. The estimated PFS rate was 93% at 12 months. Of note, four patients had discontinued treatment early due to treatment-emergent adverse events (TEAEs) and 74% of patients had any dose modification. Overall, 51% of patients experienced TEAEs of grade ≥ 3 [88]. In part C of the study, 125 patients were enrolled with a median age of 33 years. The overall CMR rate was 91% at EoT. No patients discontinued treatment early due to TRAEs [89].

BV-AVD followed by nivolumab consolidation was recently evaluated in a study of 75 patients with early-stage disease. Treatment included three cycles of BV-AVD as induction, followed by either nivolumab consolidation for iPET3-negative patients or an additional four cycles of BV-AVD and subsequent nivolumab consolidation for iPET3-positive patients. An impressive percentage of 97% of the patients achieved a negative iPET3 after BV-AVD induction with a CMR rate of 100% at EoT-PET. With a median follow-up of 22 months, there has been no event of disease progression or death in all eligible patients who received any therapy per the study protocol [90].

## 6. Novel Agents in the First-Line Treatment of Classical Hodgkin Lymphoma of the Elderly

The treatment of elderly patients with cHL remains challenging due to several limitations. Comorbidities and performance status may reduce patients’ tolerance to standard therapy or preclude a relatively intensive treatment and ultimately compromise survival. When they are eligible for ABVD and treated similarly to younger patients, elderly patients with cHL may enjoy similar or slightly inferior tumor control rates [91,92]. Patients > 60 years old are prone to chemotherapy-related toxicity with ~5% toxic death rate with ABVD [92] and are definitely ineligible for BEACOPP-escalated approaches. Elderly patients are also at much higher risk of potentially lethal bleomycin-related pulmonary toxicity. Furthermore, a fraction of elderly patients has anthracycline contraindications precluding ABVD treatment. Thus, there is a hope that the incorporation of novel agents in first-line treatment could offer the potential for improved outcomes with minimal toxicity. The safety and effectiveness of BV has been explored in various studies during the past years, either in combination with chemotherapy or as a single agent. The experience with CPIs or BV-CPI combinations remains more limited.

### 6.1. BV Combined with AVD

AVD is an appealing regimen for elderly patients due to the elimination of bleomycin and the potentially fatal pulmonary toxicity, which is much more common and more lethal in this population. BV has been evaluated with AVD under two different schedules: concurrent BV-AVD was compared with ABVD in the ECHELON-1 trial, while a sequential BV-AVD-BV program was adopted in a phase 2 trial conducted by Evens et al. [93,94].

In the ECHELON-1 trial, 186/1334 stage III/IV patients (14%) were >60 years old; 84 were randomized to BV-AVD and 102 to standard ABVD. Despite the assumption that BV-AVD could offer an advantage over ABVD in this population, in which iPET-based treatment intensification is prohibitive, this was not confirmed in the respective subgroup analysis. However, it should be noted that this analysis was not powered to detect a mPFS benefit in this subpopulation. Although toxic deaths were less with BV-AVD (3.6% vs. 5.1% with ABVD), the 2-year mPFS was similar (70.3% vs. 71.4%) [42,93]. When evaluated per investigator, there was some numerical difference in favor of BV-AVD (67.1% vs. 61.6%), which was, however, still not significant (HR 0.82, 95% CI 0.49–1.36, *p* = 0.44). Like younger patients, only 8% had a positive iPET. As in the overall population, in elderly patients, iPET also maintained its predictive role: the 5-year PFS for iPET-positive group was 40.0% vs. 25.0% for BV-AVD vs. ABVD, respectively, albeit the number of patients was very small. However, the NPV of iPET was even lower compared with younger patients, as the 5-year PFS was 71.9% vs. 64.9% for the iPET-negative patients receiving BV-AVD and ABVD, respectively [44]. Regarding toxicity, 80% of the patients required some degree of BV dose modification, including 20% BV withdrawals [93]. Neutropenia of grade ≥ 3, febrile neutropenia and PN were more frequent with BV-AVD in contrast to pulmonary toxicity, which was reported in 13% of ABVD-treated patients but was almost absent with BV-AVD [93].

Evens et al. conducted a very interesting phase 2 study of 48 elderly patients with a median age of 68 years and early unfavorable stage II or advanced-stage disease, who received BV plus AVD in a sequential approach: initially two doses of single-agent BV were administered (lead-in phase), followed by six cycles of AVD and then four doses of BV consolidation for responders [94]. The rationale of this sequential schedule was to offer early-disease control, deliver chemotherapy in patients with a potentially improved general condition and, at the same time, avoid the overlapping toxicity of BV and AVD, especially severe neutropenia or—hopefully—reduce neuropathy. Response assessment after the BV × 2 lead-in phase was mandatory only for the 22 initial patients: the ORR after BV × 2 was 82% with 36% CR. In the interim setting, following BV × 2 and AVD × 3, PET remained positive in 24% of the patients. The 2-EFS, PFS and OS were 80%, 84% and 93%. The risk of toxic death was low (1/48 patients or 2%), while the rates of febrile neutropenia and PN appeared lower compared with BV-AVD. As patient populations were different, the results of the two studies regarding efficacy and toxicity are comparatively presented in Table 4 [93,94].

### 6.2. BV Monotherapy

BV monotherapy was tested as a less toxic option for first-line therapy of cHL. As reported above, two BV infusions induced an ORR of 82% with 36% CRs in a series of 48 elderly patients with predominantly advanced disease [94]. BREVITY, a phase 2 trial with 35 patients with advanced disease or unfavorable stage II (B and/or bulk) unfit for standard chemotherapy (predominantly elderly, median age 77 years) produced similar results with an ORR of 84% and 26% CRs after the initial four BV cycles [95]. In contrast, in another small phase 2 trial of 27 elderly patients with more favorable features, the best ORR was 92%, but the CR rate (as best response) was as high as 73% [96]. A comparative summary of these trials regarding eligibility criteria, patients’ characteristics and outcomes given in Table 5. Although the CR rates were substantially different, the median PFS was 7.3 and 10.5 months and the 1-year PFS was 14% and ~35%, respectively. Thus, BV monotherapy can induce disease control in the vast majority of elderly patients with advanced disease, but CR rates are variable and generally low, while long-term PFS is clearly suboptimal. Based on these considerations, BV monotherapy can be used for transient disease control or to delay cytotoxic chemotherapy, especially in frail patients with relatively limited life expectancy, but in most cases cannot be used with a curative intent. Furthermore, severe peripheral neuropathy was rather common (23–35%) and more frequent in patients with diabetes and/or hypothyroidism and could lead to permanent BV discontinuation [95]. 

### 6.3. Anthracycline-Free BV-Chemo Combinations

Although most elderly patients can tolerate standard anthracycline therapy in referral centers, a considerable proportion has absolute or relative contraindications. Nevertheless, the relative anthracycline contraindications are not clearly defined and may be subjective. Anthracycline-free regimens are generally considered inferior to ABVD, but BV might increase their efficacy. As BV monotherapy is not a curative option for cHL, researchers have attempted to combine BV with conventional cytotoxic agents by introducing “anthracycline-free” BV-chemo combinations as a less intensive approach compared to BV-AVD in the elderly. Relevant phase 2 trials are presented in more detail in Table 5.

BV plus bendamustine is an established and highly effective combination for relapsed/refractory disease [100]. In a non-randomized study by LaCasce et al. exploring the effectiveness of BV alone, BV-dacarbazine, BV-bendamustine or BV-nivolumab, 42 elderly patients ≥ 60 years old received either BV plus dacarbazine or BV plus bendamustine. BV-dacarbazine was considered an acceptable and safe combination with all patients responding and 62% achieving a CMR as best response. The median PFS was 46.8 months. Although this is much longer than the ones achieved with BV alone, it still corresponds to a <50% PFS rate at 5 years, which remains suboptimal [97]. Twenty patients received BV-bendamustine with 100% ORR. However, nearly all patients experienced significant toxicity including grade ≥ 3 adverse events in 18 patients and serious adverse events in 13 patients. Of note, toxicity remained detrimental even when investigators reduced the dose of bendamustine from 90 to 70 mg/m^2^, leading to a treatment suspension. The median PFS was comparable to that achieved with BV-dacarbazine with a median value of 40.3 months [97]. Similarly, the HALO trial [98] aimed to explore the efficacy and tolerability of BV-bendamustine in 59 patients aged 60–80 years with advanced disease including stage IIB. With a median age of 70 years, most of the patients were frail according to the Activities of Daily Living (ADL) and Instrumental ADL scales and suffered from cardiovascular or metabolic comorbidities. After a mean follow-up of 20.6 months, the CR rate was again 63% with 2-year PFS and OS of 54% and 83%. Interestingly, 17 patients experienced CMV reactivation, 12 received valganciclovir, 3 of them interrupted treatment for this reason and 4 of them died with CMV viremia. 

Overall, these BV-chemo combinations were associated with rather high rates of grade ≥ 3 PN and frequent events of treatment discontinuation. BV-bendamustine may be an acceptable choice for older patients with advanced disease, but it should be applied with caution. Optimal bendamustine dosing still needs to be defined and patients must be closely observed for toxicity including CMV monitoring.

### 6.4. Checkpoint Inhibitor Monotherapy with or without BV for Frail or CT-Ineligible Patients

BV plus nivolumab followed by autologous stem cell transplantation is a highly active second-line regimen for relapsed/refractory cHL [101]. The combination BV-nivolumab has been assessed in two phase 2 trials, namely the previously mentioned 4-arm non-randomized trial [99] and the ACCRU trial [102], as summarized in Table 5. In the former, 20 patients ≥ 60 years old with predominantly stage III/IV (80%) and good PS (only 5% had PS ≥ 2) received the combination and achieved a best ORR of 95%. Best CR rates have not been reported and no mature PFS and OS data have been published, as the median observation is still short in the order of 19.4 months [99]. In the ACCRU trial, the patients received eight cycles of the combination with surprisingly low ORR (64%) and CR rates (52%) [102].

NIVIHO is the first trial aimed at assessing the efficacy and feasibility of nivolumab monotherapy in old and frail patients with HL, including 64 patients with a median age of 74 years, impaired PS and mostly advanced disease. Treatment consisted of six nivolumab infusions, early assessment and subsequent nivolumab monotherapy for 18 additional cycles in patients with CMR. Patients with partial metabolic response (PMR) or no response received a combination of nivolumab plus vinblastine for 18 cycles. The results were poor, as 23% of the patients died during treatment (8/15 disease- or toxicity-related). The discontinuation rate was again high, with 30% of patients withdrawn due to nivolumab-related adverse events. The ORR was 52% and only 29% of the patients achieved CR. With a median follow-up of 20.1 months, the median PFS remains suboptimal at 9.8 months [103]. 

A similar rational pembrolizumab monotherapy was assessed in a single-arm trial of 27 patients aged ≥ 65 or unfit for upfront ABVD due to comorbidities. Treatment schedule consisted of pembrolizumab 200 mg every 3 weeks for up to 35 infusions (2 years). The median age was 77 years, and 92% of patients had stages III/IV. The CMR rate was 32%. With median follow-up of 25.1 months, the 12-month and 24-month OS was 90% and 83%. The median duration of response was 10.6 months. All patients experienced adverse events and five patients discontinued treatment, but there were no treatment-associated deaths. Overall, pembrolizumab monotherapy appears a feasible and safe option for frail patients [104]. 

N-AVD could also be an appealing option for elderly patients based on the recent results of the SWOG S1826 study, which demonstrated superior efficacy with a better safety profile than BV-AVD. With 93 advanced-stage patients >60 years old randomized between N-AVD and BV-AVD, the hazard ratio for PFS was 0.27 (95% CI 0.10–0.76, *p* = 0.013) in favor of N-AVD vs. 0.56 and 0.48 for the age groups 18–60 and 12–17 years old (presented in ASCO 2023 and ICML 2023) [45]. These impressive results make N-AVD the most attractive approach for elderly patients suitable for anthracycline-based chemotherapy. N-AVD was also administered in a phase 2 trial of 33 patients ≥ 60 years old, all of whom received six cycles. Of the 33 patients, 41% were ≥70 years old and 78% had advanced-stage disease. The CR rate at EoT was 97%. With a median follow-up of 37 months, the 2-year PFS and OS were 86.2% and 96.4%, respectively. Although 41% of the patients had grade ≥ 3 treatment-related adverse events, only two patients stopped treatment [105]. Overall, N-AVD was highly effective and well-tolerated, but more real-life studies are needed in order to assess its feasibility in elderly and frail populations in relation to geriatric assessment. 

Generally, BV monotherapy and combinations with non-anthracycline regimens appear to be inferior to BV-AVD and its variants in elderly patients. However, N-AVD appears superior to BV-AVD in advanced-stage elderly patients. For patients ineligible for ABVD, BV-AVD or N-AVD, these approaches can provide reasonable alternatives, although their value compared with older chemotherapeutic approaches such as MOPP (mechlorethamine, vincristine, procarbazine, prednisone), ChlVPP (chlorambucil, vinblastine, procarbazine, prednisone), or other regimens is uncertain. Although responses are short-lived, the introduction of CPIs may also offer another safe option for patients unsuitable for chemotherapy.

## 7. Ongoing and Forthcoming Randomized Trials

The scientific interest of incorporating novel agents in the first-line treatment of cHL is increasing. Apart from promising phase 2 studies, large and randomized trials are required to establish their benefit in this setting, define their precise role and obtain regulatory approvals. The SWOG S1826 study has already suggested the superiority of N-AVD over BV-AVD in advanced cHL, while BEACOPP escalated has been overcome by BreCADD, based on the results of HD21, as described above. The long-term results of these trials are eagerly awaited. As the rate of primary refractoriness and early relapses is reduced, the occurrence of late and very late relapses may become a new field of intense investigation [106]. 

INDIE is a trial of the GHSG currently in progress, which attempts to incorporate an immunotherapy-based individualized approach in intermediate-stage (early unfavorable) disease using tislelizumab, another anti-PD-1 monoclonal antibody [107,108]. All patients will receive two cycles of tislelizumab monotherapy followed by an iPET. Patients will continue with four cycles of tislelizumab plus AVD (T-AVD) or four cycles of tislelizumab alone in case of positive or negative iPET, respectively. Patients with a positive EoT-PET will additionally receive 30Gy of involved-site radiation therapy (ISRT) [108]. As tislelizumab is a less expensive CPI, monotherapy or T-AVD, it might be a cost-effective option for the first-line therapy of cHL if the results are satisfactory.

RADAR is another phase 3 trial in progress which will evaluate the efficacy of ABVD vs. BV-AVD with or without ISRT. The eligibility criteria are the same as that of the RAPID trial [10]: (1) age 16 to 69 years; (2) stage IA/IIA supradiaphragmatic disease; and (3) absence of bulky mass. Patients will be randomized to receive either ABVD or BV-AVD, and iPET2 will determine further treatment according to the D5PSS. Patients with D5PSS-1-3 will receive three cycles in total ± an individualized RT. D5PSS-4 patients will receive four cycles + 30 Gy ISRT, whereas D5PSS-5 patients represent treatment failure and will continue with the treating physician’s choice. The primary endpoint will be PFS, and the trial will have 85% power to show an improvement from 90% to 95% PFS at 3 years (HR of 0.49) with 2-sided 5% alpha [109].

Finally, RAFTING is a prospective, phase 2 single-cohort international study aimed at exploring the effectiveness of a risk-adapted treatment strategy in non-bulky stage I/IIA favorable HL, based on the risk of chemotherapy failure assessed on a single-patient basis and in a personalized medicine design. The primary endpoint is a 3-year PFS ≥ 90% in low-risk patients (see below) treated with ABVD chemotherapy alone. Treatment intensity is tailored to three classes of patients with a different risk of treatment failure, depending on (a) the modified EORTC criteria (m-EORTC), in which classical bulk definition is replaced by a large nodal mass (LNM, defined by the largest diameter > 5 cm in CT or PET/CT scans), (b) TMTV assessed at baseline and (c) the PET2 result. The risk-stratified treatment is the following: Group 1 (Low risk): patients with a negative PET2 and low TMTV, treated with 2–4 ABVD cycles (depending on the absence or presence of at least one m-EORTC criterion), addressed, once in CR, to a trimonthly cell-free tumor DNA (ctDNA) assay [110], in order to detect as early as possible an impending eHL relapse; Group 2: patients belonging to group 1 relapsing with the pattern of “Limited Relapse” (no more than three new nodal areas), treated with involved-node radiotherapy (INRT) at the dose of 36 Gy, and Nivolumab-maintenance at the dose of 240 mg twice in a month for one year; Group 3 (high Risk): PET2 positive (Group 3a), and/or a high bMTV (Group 3b) patients treated with ABVD × 4, INRT, (20 or 30 Gy) and Nivolumab maintenance as in Group 2 [111].

## 8. Conclusions

It is very likely that the future of cHL treatment will be dominated by the introduction of novel agents in the first-line treatment of the disease, through various schedules and combinations. Both BV and CPIs have several benefits and limitations, while their impact in PET-adapted approaches is still unknown. In advanced cHL, both N-AVD and BreCADD will probably dominate soon, especially if regulatory approval is granted. How they compare with each other is unclear, but the favorable toxicity profile makes N-AVD very attractive. PET-adapted therapy may also be affected in advanced disease if N-AVD is adopted.

In localized stages, the incorporation of novel agents in the first line will require further clinical trials and time. While novel agents spare the classical toxicity related to conventional chemotherapy, specific forms of toxicity of new non-chemotherapy drugs indeed exist and should not be overlooked. 

Furthermore, very late relapses remain an issue in HL and cannot be captured by the early results of the aforementioned trials [112,113,114]. More mature data are needed in the long term to draw robust conclusions about the efficacy of novel agents and the true benefit of their early incorporation in first-line treatment.

## Figures and Tables

**Table 1:** Summary of initial and updated efficacy and toxicity results of ECHELON-1 and SWOG S1826 trials.

Patients’ Characteristics and Key Outcome and Toxicity Measures	ECHELON-1		SWOG S1826	
	ABVD	BV-AVD	BV-AVD	N-AVD
**Patients (Ν) and patient characteristics** [42,44,45]	670	664	487	489
Age (median (range))	37 (18–83)	35 (18–82)	26 (12–81)	27 (12–83)
Stage IV (%)	63	64	65	62
IPS 4–7 (%)	26	25	32	32
**Outcome measures** [42,43,44,45]				
*All patients*				
1-year PFS (%) *	81.4	88.6	86 ^m^	94 ^m^
2-year mPFS per IRC (%) (primary endpoint) [42]	77.2 ^a^	82.1 ^a^	ND	ND
6-year PFS per INV (%) [43]	74.5 ^b^	82.3 ^b^	NYA	NYA
6-year OS (%) [43]	89.4 ^c^	93.9 ^c^	NYA	NYA
*Stage IV or III subgroup*				
2-year mPFS per IRC, Stage IV (%) [42]	74.9 ^d^	82.0 ^d^	ND	ND
6-year PFS per INV, stage III (%) [43]	NR ^e^	NR ^e^	NYA	NYA
6-year PFS per INV, stage IV (%) [43]	NR ^f^	NR ^f^	NYA	NYA
6-year OS, stage III (%) [43]	NR ^g^	NR ^g^	NYA	NYA
6-year OS, stage IV (%) [43]	NR ^h^	NR ^h^	NYA	NYA
*Interim PET negative status* [44]	n = 578	n = 588		
5-year PFS per INV, all patients (%)	78.9 ^i^	84.9 ^i^	NYA	NYA
5-year PFS per INV, <60 years old (%)	81.5 ^j^	86.6 ^j^	NYA	NYA
*Interim PET positive status* [44]	n = 58	n = 47		
5-year PFS per INV, all patients (%)	45.9 ^k^	60.6 ^k^	NYA	NYA
5-year PFS per INV, <60 years old (%)	49.3 ^l^	63.1 ^l^	NYA	NYA
**Toxicity** [42,45]				
Toxic deaths, all patients [N (%)]	13 (1.9)	9 (1.4)	NR	1 (<%)
Hospitalization, all patients (%)	28	37	NR	NR
Peripheral sensory neuropathy, all patients, all grades (%)	17	29	55	29
Peripheral sensory neuropathy, all patients, grade ≥ 3 (%)	<1	5	8	1
Peripheral motor neuropathy, all patients, all grades (%)	4	11	7	4
Febrile neutropenia, all patients (%)	8	19	7	5
Febrile neutropenia, elderly (%)	17	37	-	-

ABVD = Adriamycin, Bleomycin, Vinblastine and Dacarbazine; BV-AVD = Brentuximab Vedotin, Adriamycin, Vinblastine and Dacarbazine; N-AVD = Nivolumab, Adriamycin, Vinblastine and Dacarbazine; NR = Not Reported; ND = Not Defined; NYA = Not Yet Available; IPS = International Prognostic Score; per IRC = per Independent Review Committee; per INV = per investigator; PFS = Progression Free Survival; mPFS = modified Progression Free Survival; OS = overall survival. * Approximated from published survival curves [42]. Hazard Ratios (95%CI)/*p*-values: ^a^ 0.77 (0.60–0.98)/*p* = 0.03; ^b^ 0.68 (0.53–0.86)/*p* = 0.0017; ^c^ 0.59 (0.40–0.88)/*p* = 0.009; ^d^ 0.71 (0.53–0.96)/NR; ^e^ 0.60 (0.39–0.93)/NR; ^f^ 0.72(0.53-0.96)/NR; ^g^ 0.86 (0.45–1.65)/NR; ^h^ 0.48 (0.29–0.80)/NR; ^i^ 0.66 (0.50–0.88)/*p* = 0.0035; ^j^ 0.68 (0.49–0.93)/*p* = 0.014; ^k^ 0.70 (0.39–1.26)/*p* = 0.23; ^l^ 0.70 (0.37–1.33)/*p* = 0.27; ^m^ 0.48 (0.27–0.87)/*p* = 0.0005.

**Table 2:** Clinical trials incorporating brentuximab vedotin in the first-line treatment of early-stage classical Hodgkin lymphoma.

	Abramson J.S. et al., 2019 [54]	Abramson J.S. et al., 2023 [55]	Park S.I. et al., 2020 [56]	Kumar A. et al., 2021 [57]	Fornecker L.M. et al., 2022 [58]
**Patients (N)**	36	34	39	116	170
**Eligible stages**	I/II non-bulky ^◊^	I/II non-bulky ^◊^	I/II non-bulky ^±^	Stage I, II unfavorable ^¶^	Stage I/II unfavorable ^&^
**Initial treatment**	BV-AVD × 2	BV-AD × 2	ABVD × 2	BV-AVD × 2	BV-AVD × 2 vs. ABVD × 2 (113 vs. 57 patients)
**Further treatment**				BV-AVD × 4 (#4) (*not PET2 directed*)	BV-AVD × 2 (#4) vs. ABVD × 2 (#4) (*not PET2 directed*)
PET2−	BV-AVD × 2 (#4)	BV-AD × 2 (#4)	F: BV × 6 U: ABVD × 2 (#4) + BV × 6		
PET2+	BV-AVD × 4 (#6)	BV-AD × 4 (#6)	F: ABVD × 2 (#4) + BV × 6 U: ABVD × 4 (#6) + BV × 6		
**PET2+ definition**	DS 4–5	DS 4–5	DS 3–5	DS 4–5	DS 4–5
**PET2+ patients (N)**	0	6%	28%	13%	17.7% vs. 24.6%
**RT (% of patients)**	No	No	Yes, for EoT PET+ (5%)	Yes, for cohorts 1–3	Yes, for all patients after treatment (30 Gy)
**PFS**	3-year: 94%	5-year: 91%	3-year: 92% (*100% for patients* *with EoT PET−*)	2-year PFS: 93.1%, 96.6%, 89.7%, 96.6% for cohorts 1–4	2-year: 97.3% vs. 92.6% *For PET2−: 97.8% vs. 97.7%* *For PET2+: 93.8 vs. 71.6%*
**OS**	3-year: 97%	5-year: 96%	3-year: 97% (*100% for patients* *with EoT PET−*)	2-year: 99% in the whole population	NR
**Toxicity** **(% of patients)**					
PN grade ≥ 3	24%	0% *	2.5%	3%	3 vs. 2%
FN	29%	0% **	NR	8%	8% vs. 6%
**EoT PET-negative** **(% of patients)**	91.2%	97%	95%	93%, 100%, 93%, and 97% for cohorts 1–4	88% vs. 77%

PET2: Positron Emission Tomography after 2 cycles of treatment, #: total number of treatment cycles, RT: Radiotherapy, PFS: Progression Free Survival, OS: Overall Survival, PN: Peripheral Neuropathy, FN: Febrile Neutropenia, EoT: End of Treatment, BV: Brentuximab Vedotin, ABVD: Doxorubicine—Bleomycin—Vinblastine—Dacarbazine, DS: Deauville Score, NR: Not Reported. ^&^ according to EORTC criteria. ^◊^ defined as lymphoid lesion > 10 cm in maximal dimension. * Only 3/34 patients developed grade 2. ** No preemptive use of Granulocyte colony-stimulating factor (G-CSF). ^±^ defined as lymphoid lesion >7.5 cm in maximal dimension. ^¶^ according to GHSG criteria. For cohorts 2–4, “bulky” was defined as >7.5 cm (Memorial Sloan Kettering definition). Note that 23% of patients had stage IIBX.

**Table 3:** Clinical trials incorporating checkpoint inhibitors in established chemotherapy regimens in the first-line treatment of classical Hodgkin lymphoma.

	Ramchandren R. et al., CHECKMATE-205, Arm D, 2019 [71]	Bröckelmann P.J. et al., NIVAHL, 2023 [72]	Allen P.B. et al., 2022 [73]	Lynch E.C. et al., 2023 [74]
**Patients (N)**	51	109	30	30
**Eligible stages**	Advanced * (III/IV, IIBX/E)	I/II unfavorable *	III/IV, I/II unfavorable ^¶¶^	All
**CPI lead-in phase**	Nivo × 4	Nivo × 4 (Only Arm B)	Pembro × 3	no
**Response to CPI monotherapy [ORR (CR)]**	69% (18%)	NR (51%)	NR (37% + 23%) ***	NA
**Overall Treatment Schedule**	Nivo × 4 followed by AVD × 6	*Arm A*: Nivo-AVD × 4 + 30 Gy ISRT *Arm B*: Nivo × 4 → Nivo-AVD × 2 → AVD × 2 + 30 Gy ISRT	Pembro × 3 followed by AVD × 4–6 **	Pembro-AVD × 2–6 **
**CR rate at EoT**	80%	*Arm A*: 83% ^¶^ *Arm B*: 84% ^¶^	100% (at EoT)	78% (at EoT)
**PFS**	92% (9-month)	*Arm A*: 100% (3-year) *Arm B*: 98% (3-year)	100% (2-year)	97% (2-year)
**OS**	98% (9-month)	3-year OS 100% in both arms	100% (2-year)	100% (2-year)

CPI = Checkpoint inhibitor; AVD = Adriamycin, Vinblastine and Dacarbazine; ORR = overall response rate; CR = complete remission; EoT = end of treatment; PFS = progression free survival; OS = overall survival; NR = not reported; ISRT = involved site radiotherapy; NA = not applicable. * GHSG criteria; ** number of cycles determined according to stage (and bulk in Allen et al. [69]); ^¶^ at the end of systemic therapy—prior to radiotherapy; ^¶¶^ NCCN criteria; *** 37% CR plus 23% near CR (>90% reduction in metabolic tumor volume).

**Table 4:** Comparison of sequential vs. concomitant BV-AVD in elderly patients with unfavorable or advanced cHL.

Patients’ Characteristics and Outcomes	BV-AVD (Sequential Regimen) [94]	BV-AVD (Concomitant Regimen) [93]	ABVD Comparator [93]
**Patients (N)**	48	84	102
**Age [years, median (range)]**	69 (60–88)	68 (60–82)	66 (60–83)
**ECOG PS ≥ 2**	19%	12%	10%
**Ann Arbor Stage**			
II	19%	0%	0%
III	37%	37%	34%
IV	44%	61%	66%
**Efficacy**			
Response to BV × 2 [ORR (CR)]	82% (36%)	NA	NA
Interim PET positive	24% (10/42 pts)	20%	18%
EoT PET-negative	90% (38/42 pts)	71%	74%
**2-year PFS**	84%	70.3% *	71.4% *
**5-year PFS**	NA	67.1%	61.6%
**2-year OS**	93%	NA	NA
**Toxicity**			
TRM	2%	3.6%	5.1%
Neutropenia, grade ≥ 3	44%	70%	59%
Febrile neutropenia, grade ≥ 3	8%	37%	17%
Peripheral neuropathy			
any grade	NA	65%	43%
grade ≥ 3	4%	18%	3%
grade 2	27%	37%	16%
resolution	69%	80%	83%
Pulmonary toxicity, any grade	NA	2%	13%

ECOG: Eastern Cooperative Oncology Group, BV: Brentuximab Vedotin, PET: positron emission tomography, PFS: progression-free survival, OS: overall survival, TRM: treatment-related mortality, ORR: overall response rate, CR: complete response, EoT: end of treatment, NA: not available, ABVD: Doxorubicin, Bleomycin, Vinblastine, Dacarbazine AVD: Doxorubicin, Vinblastine, Dacarbazine. * modified progression-free survival (modified PFS).

**Table 5:** Comparative presentation of patient characteristics, outcomes and toxicity with BV monotherapy, anthracycline-free BV chemotherapy combinations or BV-nivolumab combinations in elderly patients with cHL.

Patients’ Characteristics, Outcome, and Toxicity	BV Monotherapy (BREVITY) [95]	BV Monotherapy [96]	BV-Dacarbazine [97]	BV-Bendamustine [97]	BV-Bendamustine (HALO) [98]	BV-Nivolumab [99]
**Patients (total, N)**	35	27	22	20	60	20
**Patients (evaluable, N)**	31	26	19	17	59	19
**Eligibility criteria**	Stage IIBX/III/IV unfit for standard CT *	≥60 years old	≥60 years old	≥60 years old	Stage IIB/III/IV ≥60 years old	≥60 years old
**Follow-up time (median)**	3-years	59.4 months	58.6 months	51.3 months	20.6 months	19.4 months
**Treatment cycles**	up to 16	up to 16	up to 12 plus BV ≥ 4	up to 6 plus BV ≥ 10	up to 6	NA
**Age** **[median (IQR or range)]**	77 (72–82)	78 (64–92)	69 (62–88)	75 (63–86)	70.32 (62–79)	72 (NR–NR)
**Ann Arbor Stage III, IV**	80%	63%	68%	75%	80%	80%
**ECOG PS ≥ 2**	48%	22%	32%	20%	10%	5%
**B-symptoms**	71%	22%	29%	41%	68%	NA
**CIRS** **[median (IQR or range)]**	5 (4, 7)	NA	NA	NA	NA	NA
**TRM**	0.35%	0%	0%	0%	NA	0%
**ORR**	84% **	92% ***	100% ***	100% ***	63%	95%
**CMR**	26% **	73% ***	62% ***	88% ***	80.36%	NA
**PFS**						
Median	7.3 months	10.5 months	46.8 months	40.3 months	NR	NR
1-year	14%	~35%	NA	NA	84%	NA
2-year	7%	~30% ^+^	NA	NA	54%	NA
**OS**						
Median	19.5 months	77.5 months	64 months	46.9 months	83%	NR
1-year	73%	NA	NA	NA	97%	NA
2-year	42%	NA	NA	NA	83%	NA
**Peripheral neuropathy** **grade ≥ 3**	NR ^++^	30% ^&^	26%	20%	0%	35%
**Discontinuation rate**	29%	42%	40%	40%	30.5%	30%

CT: chemotherapy, ECOG: Eastern Cooperative Oncology Group, CIRS: Cumulative Illness Rating Scale-Geriatric, TRM: treatment-related mortality, ORR: overall response rate, CMR: complete metabolic response, PFS: progression-free survival, OS: overall survival, BV: Brentuximab Vedotin, NA: not available, NR: not reached. * unfit for chemotherapy were patients including one of the following: impaired cardiac function with ejection fraction of <50% assessed by echocardiogram or MUGA scan or of ≥50% but in the presence of significant comorbidities or cardiac risk factors such as diabetes mellitus, hypertension, peripheral vascular disease, ischemic heart disease, previous myocardial infarction, obesity, stroke or transient ischaemic attacks (TIA) that make anthracycline-containing chemotherapy inadvisable or with clinically determined heart failure (NYHA II and III) or with impaired respiratory function with DLCO and/or FVC/FEV1 ratio < 75% of predicted or ECOG PS score 1–3 in patients aged ≥60 years. ** The percentage reflects response rate at cycle 4. *** The percentage reflects the best response rate. ^+^ at 22 months. ^++^ 23% discontinued due to peripheral neuropathy. ^&^ 46% vs. 14% of those without or with diabetes and/or hypothyroidism.

## Data Availability

Not applicable.
